# Harnessing the Power of Plasmonics for *in Vitro* and *in Vivo* Biosensing

**DOI:** 10.1021/acsphotonics.4c01657

**Published:** 2025-02-17

**Authors:** Ediz Kaan Herkert, Maria F. Garcia-Parajo

**Affiliations:** † ICFO - Institut de Ciencies Fotoniques, The Barcelona Institute of Science and Technology, Castelldefels 08860 (Barcelona), Spain; ‡ ICREA-Catalan Institute for Research and Advanced Studies, Pg. Lluis Companys 23, Barcelona 08010, Spain

**Keywords:** localized surface plasmon resonances, plasmonic enhancement, multiresonant and broadband plasmonics, plasmonic metamaterials, biosensors for disease diagnosis in complex biofluids, multiplexed bioanalyte detection

## Abstract

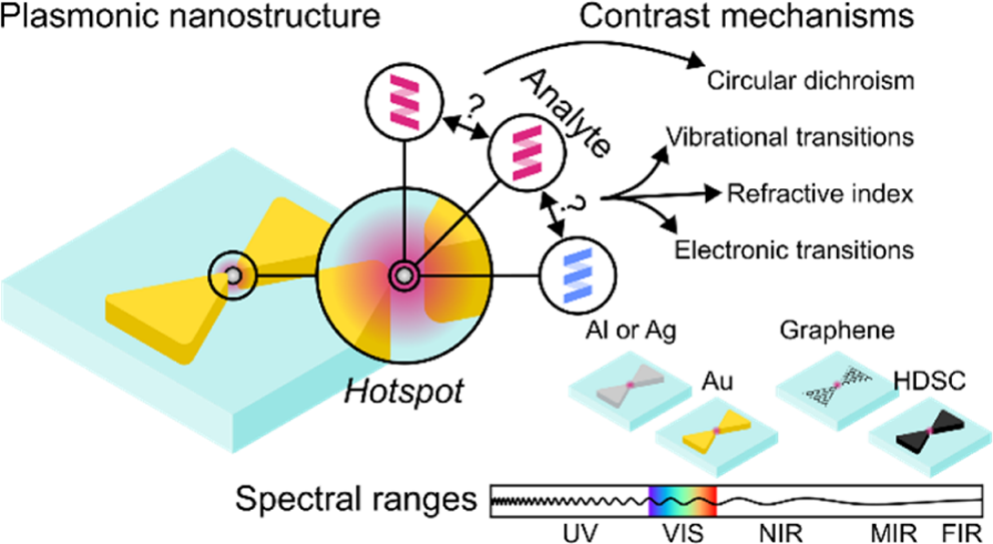

Plasmonic nanostructures exhibit localized surface plasmon resonances due to collective oscillation of conducting electrons that can be tuned by modulating the nanostructure size, shape, material composition, and local dielectric environment. The strong field confinement and enhancement provided by plasmonic nanostructures have been exploited over the years to enhance the sensitivity for analyte detection down to the single-molecule level, rendering these devices as potentially outstanding biosensors. Here, we summarize methods to detect biological analytes *in vitro* and *in living cells*, with a focus on plasmon-enhanced fluorescence, Raman scattering, infrared absorption, circular dichroism, and refractive index sensing. Given the tremendous advances in the field, we concentrate on a few recent examples toward biosensing under highly challenging detection conditions, including clinically relevant biomarkers in body fluids and nascent applications in living cells and *in vivo*. These emerging platforms serve as inspiration for exploring future directions of nanoplasmonics that can be further harnessed to advance real-world biosensing applications.

## Introduction

Optical biosensing and bioimaging methods provide powerful noninvasive ways to detect various biomolecular analytes like pathogens, proteins, drugs, or antibodies establishing them as indispensable tools in clinical diagnosis, environmental monitoring and, life sciences applications.^1^ However, the sensitivity provided by standard optical sensing methods is often insufficient to detect small amounts of analytes typically associated with many pathological conditions. Moreover, in the field of bioimaging, standard optical microscopy approaches are compromised by the diffraction limit of light that restricts the resolution to about half the wavelength at best. Because of this, a wide range of nanoplasmonic biosensing and -imaging platforms have emerged in recent years that provide superior sensitivity and surpass the diffraction limit.^2−4^

Plasmonic nanostructures are nanoscale metallic particles that can evanescently confine external optical fields in subdiffraction volumes (*V* ≪ λ^3^) in their immediate vicinity. The intensity of these tightly confined fields can exceed the intensity of the external field by orders of magnitude due to the optical excitation of localized surface plasmon resonances (LSPRs) within the plasmonic nanostructures. The strong field confinement and enhancement provided by plasmonic nanostructures are the two key factors that enhance the sensitivity for analyte detection above those of standard optical biosensing and -imaging schemes.

When evaluating biosensor concepts, several criteria in terms of performance, operation, and cost come into play. Performance considerations include sensitivity, selectivity, specificity, and throughput, while operational factors include portability, reusability, and ease of use. Plasmonic platforms are commonly associated with improved performance, however at the expense of increased fabrication costs and operational complexity. Yet, plasmonic nanostructures have the potential to enable more cost-effective and compact platforms as the provided performance gains allow for the use of less expensive and complex light sources and detectors,^5^ as well as requiring lower volumes and/or concentrations of the analytes to be detected. The growing availability of cost-effective fabrication methods and suitable surface functionalization strategies is further improving the competitiveness of nanoplasmonics in biosensing and -imaging applications.^6−11^

This review offers insights into promising future directions of nanoplasmonic biosensing based on the latest advancements in the field. It summarizes common methods to detect biological analytes *in vitro* and in living cells, and outlines how their sensitivity scales with the field enhancement in the subwavelength plasmonic *hotspot*. Furthermore, it compares emerging nanoplasmonic platforms designed for biological applications with a strong focus on their improved performance in terms of sensitivity, selectivity and throughout. These emerging platforms serve as inspiration for exploring how the potential of nanoplasmonics can be further harnessed to advance real-world biosensing applications.

## NANOPLASMONICS FOR ENHANCED ANALYTE DETECTION

**Figure 1:**
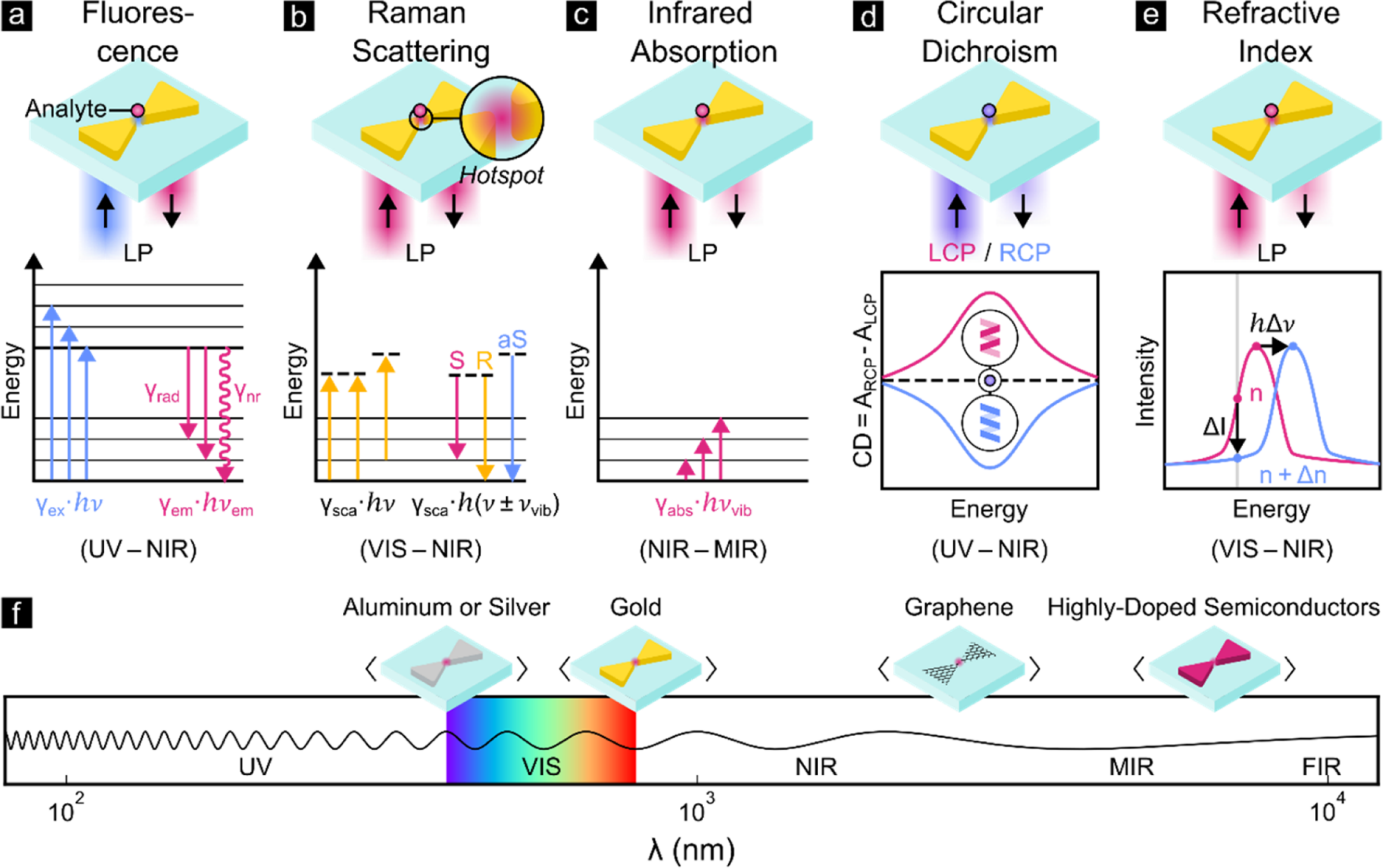
Plasmonic nanostructures can improve the detection of biological analytes using a range of detection methods (a–e) across a broad spectral range (f). (a) Plasmon-enhanced fluorescence spectroscopy increases the excitation rate γ_ex_ of a fluorescently labeled analyte by enhancing the excitation intensity in the *hotspot* as well as the emission rate γ_em_ of the analyte by modifying the quantum efficiency η_F_ = γ_rad_/(γ_rad_ + γ_nr_) depending on the rates of radiative γ_rad_ and nonradiative γ_nr_ decays. The emitted photon of energy *hν*_em_ is typically red-shifted. (b) Plasmon- or surface-enhanced Raman scattering (SERS) detects characteristic vibrational energies *hν*_vib_ via elastic Rayleigh (R), inelastic Stokes (S), and inelastic anti-Stokes (aS) scattering, with the signals γ_sca_ scaling quadratically with the plasmonic *hotspot* intensity. (c) Plasmon- or surface-enhanced infrared absorption (SEIRA) spectroscopy increases vibrational absorption rates γ_abs_ in the plasmonic *hotspot*. (d) Plasmon-enhanced circular dichroism (CD) amplifies the differential absorption of left circularly polarized (LCP) and right circularly polarized (RCP) (*A*_LCP_ and *A*_RCP_) light from chiral analytes. (e) Refractive index sensing exploits the LSPR line shape sensitivity to the environment’s refractive index, detecting analytes via induced (i) LSPR frequency shifts Δν, (ii) monochromatic intensity shifts Δ*I*, or colorimetric changes of the nanoplasmonic surface. (f) Common plasmonic materials include aluminum (UV and VIS), silver (VIS), gold (VIS and NIR), and more recently also graphene and highly doped semiconductors, which offer tunable LSPRs from the NIR to FIR. LP denotes linearly polarized excitation.

Biological analytes exhibit unique properties such as electronic and vibrational transitions, chirality, or their refractive index, which can be optically probed for their unambiguous detection. Figure 1a–e shows common detection methods to selectively differentiate biological analytes with enhanced sensitivity using nanoplasmonic platforms. These detection methods include (a) fluorescence spectroscopy^12−30^ (electronic transitions), (b) Raman scattering^31−49^ and (c) infrared absorption spectroscopy^50−62^ (vibrational transitions), (d) circular dichroism spectroscopy (chirality),^63−72^ or (e) refractive index sensing.^73−86^ They primarily benefit from the capability of plasmonic nanostructures to locally amplify the incident field intensity. This amplification is described by the intensity enhancement at a given photon energy *hv*
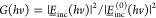
1which compares the enhanced electric field intensity |*E̲*_inc_(*hv*)|^2^ in the presence of plasmonic nanostructures to the field intensity |*E̲*_inc_^(0)^(*hv*)|^2^ without them. The intensity enhancement primarily occurs within a subdiffraction volume–the so-called plasmonic *hotspot*. Plasmonic *hotspots* are regions of high field intensity that are concentrated around the tips of plasmonic nanostructures (see Figure 1b) and arise when the wavelength of an incident field matches the LSPR wavelength. Precise control over the spectral LSPR position and the wavelength of the incident light is therefore essential to detect analytes in the plasmonic *hotspot* with high sensitivity. In addition, careful considerations should be placed when designing the sizes of the *hotspot* regions as they need to match those of the analyte to be probed. Indeed, although smaller hotpots provide higher enhancements and nanometric field confinement, some analytes such as antibodies, viruses, exosomes, etc., could be larger than the designed hotspot, resulting in inhomogeneous excitation of the analyte.^4^ Moreover, the physical position of the analyte and its dipolar orientation within the hotspot are crucial for efficient detection as the near-field intensities emanating at the *hotspot* are highly polarized and localized. Multiple strategies have been proposed over the years to maximize the interaction of the analyte within the hotspot near-field excitation and are extensively discussed elsewhere.^4,6,17,49^

The LSPR is commonly tuned by controlling the material, size, or shape of the plasmonic nanostructures.^87−89^ The material choice is particularly decisive as achieving high enhancement factors requires an efficient LSPR excitation and therefore metals with low absorption losses in the desired spectral range. Figure 1a–e depicts the typical spectral ranges of the detection methods that routinely range from the visible regime (λ > 470 nm) for fluorescence spectroscopy^90^ to λ = 10 μm for infrared absorption spectroscopy.^54^

The need to cover a wide spectral range has brought forward various materials that provide strong LSPRs in different spectral regimes. Figure 1f depicts typical spectral regions of gold, silver, aluminum, graphene, and highly doped semiconductors, which are established plasmonic materials. Gold is the most prevalent material due to its chemical stability and ability to support LSPRs from the red visible (VIS)^13^ to the mid infrared (MIR)^61^ wavelength range. Silver and aluminum are commonly used across the VIS,^74,91^ with aluminum even sustaining LSPRs in the deep ultraviolet (UV).^16^ More recently, graphene^92^ and highly doped semiconductors^93^ have emerged as alternative materials in the MIR that provide additional control over their optical properties by manipulating their charge carrier density *n*_e_ through electrostatic gating and doping, respectively. The charge carrier density is critical, as it determines the bulk plasma wavelength  of a material that dictates the lowest wavelength at which the free electrons within a material can still effectively respond to the oscillations of the incident field and thus support LSPRs.^94^ This highlights the importance of considering the bulk plasma wavelength and the absorption losses of a material to identify suitable plasmonic materials for a desired wavelength range and consequently a chosen detection method.

The following sections briefly explore the spectral ranges, capabilities, and requirements of the optical detection methods summarized in Figure 1a–e. We discuss how their sensitivity scales with the intensity enhancement *G* in the plasmonic *hotspot* and which plasmonic materials are commonly employed for the respective detection method. Based on these findings, the main considerations in the design of nanoplasmonic platforms for the detection of analytes are outlined for each detection method.

### Plasmon-Enhanced Fluorescence Spectroscopy

Fluorescent molecules can absorb light of a specific wavelength (typically in the visible) and reemit it at a typically longer wavelength. This phenomenon is illustrated in Figure 1a and is caused by transitions between the electronic ground state and higher excited states in the fluorescent molecules. The large spectral Stokes shift between the absorbed and emitted wavelengths can be effectively used as a contrast mechanism for the detection of biological analytes. The main advantage of fluorescence spectroscopy is that fluorescent molecules absorb relatively large powers
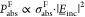
2for given incident fields, *E̲*_inc_, which result from their high fluorescence absorption cross sections σ_abs_^F^ ≈ 10^–16^ cm^2^.^95^ Moreover, the quantum efficiency for fluorescence emission can be close to unity, and a huge palette of different chromophores (including fluorescent proteins) is readily available nowadays.^96,97^ These properties have established fluorescence spectroscopy as a widely used technique in biosensing and -imaging applications.^98,99^ Fluorescence absorption and emission can be enhanced by plasmonic nanostructures through two mechanisms. First, the intensity of the incident field, *E̲*_inc_, is enhanced in the plasmonic *hotspot* leading to a stronger fluorescence absorption. Second, the quantum efficiency, η_F_, of the fluorescent molecule is modified through the quantum mechanical *Purcell* effect that alters radiative and nonradiative decay rates, γ_rad_ and γ_nr_, respectively. The fluorescence emission enhancement *G*_F_ is given by^100^
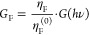
3with η_F_^(0)^ being the intrinsic quantum efficiency of the molecule in the absence of the plasmonic nanostructure, and where *G(hν)* corresponds to the intensity enhancement at the electronic absorption energies as defined in Equation 1. The fluorescence emission enhancement *G*_F_ commonly falls in the range of *G*_F_ = 10^1^–10^3^ depending on the spectral range and the intrinsic quantum efficiency, η_F_^(0)^, of the fluorescent molecule. Notably, the quantum efficiency can both be decreased or increased by plasmonic nanostructures. Typically, the quantum efficiency of inefficient fluorophores (η_F_^(0)^ ≪ 1) increases with the Purcell factor and decreases for efficient fluorophores (η_F_^(0)^ ≈ 1) because of additional metal-induced losses.^101^ Extremely narrow plasmonic cavities below 10–20 nm lead to considerable metal-induced losses and are therefore unsuitable for fluorescence applications despite their very high intensity enhancements.^102^ Materials like gold, silver, and aluminum that provide LSPRs in the absorption and emission window of most fluorescent molecules (UV to NIR) are widely used for plasmon-enhanced fluorescence applications. Plasmon-enhanced fluorescence spectroscopy stands out for combining the high fluorescence absorption cross-section of fluorescent molecules with the fluorescence emission enhancement offered by plasmonic nanostructures. This combination enables the study of analytes with exceptional sensitivity down to the single-molecule level and facilitates the label-free detection of weakly autofluorescent molecules.^16^

Nevertheless, it is important to mention that fluorescence also bears intrinsic limitations that include photobleaching (i.e., loss of photon emission), broad absorption and emission spectra that limits the number of dyes can be simultaneously employed for probing different analytes due to spectral overlap, and the inherent need to label the desired biomolecules which can affect the natural properties of the biomolecules to be detected. Increasing the photostability of organic fluorescence dyes, relying on different emitter materials (such as quantum dots or carbon nanotubes that possess narrower emission spectra as compared to organic dyes) and/or developing strategies that rely on the intrinsic autofluorescence of biomolecules, are some of the approaches being currently pursued by the community to overcome the main limitations of fluorescence spectroscopy.

### Plasmon-Enhanced Raman Scattering

Raman spectroscopy is a technique used to analyze the vibrational states of a molecule through scattering of incident photons. Figure 1b illustrates that photons can be scattered from a molecule via elastic Rayleigh scattering (R), inelastic Stokes scattering (S), or inelastic anti-Stokes scattering (aS) by exciting virtual states (dashed lines). Stokes and anti-Stokes scattering provide chemical information about the molecule as the scattered photons either lose (Stokes) or gain (anti-Stokes) energy by exciting vibrational modes with energy *hν*_vib_. These energetic Raman shifts Δ*E* = *h*(ν ± ν_vib_) can be detected spectroscopically and are characteristic of different chemical bonds within a molecule, enabling its label-free identification. The power scattered by a single molecule
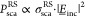
4depends on the Raman scattering cross-section σ_sca_^RS^ and the intensity of the incident electric field, *E̲*_inc_.^3^ Raman scattering is extremely weak as the single-molecule scattering cross sections are only around σ_sca_^RS^ ≈ 10^–30^ cm^2^ and thus almost 15 orders of magnitude below typical fluorescence absorption cross sections.^103^ The spectral fingerprint region between λ = 6.67–20 μm contains a series of characteristic vibrational transitions and is thus particularly suitable for the identification of analytes. Plasmonic nanostructures can strongly amplify Raman scattering in a process called surface-enhanced Raman scattering (SERS). The amplification occurs through the chemical enhancement of the scattering cross-section and the electromagnetic field enhancement of the incident electric field through the excitation of LSPRs.^47^ The SERS enhancement is described by the equation^104^
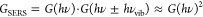
5where *G*(*hν*) and *G*(*hν* ± *hν*_vib_) are the plasmonic intensity enhancements at the incident and scattered photon energies, respectively. The intensity enhancements are approximately equal at both energies as the Raman shifts are typically very small compared to the LSPR line width. The additional chemical enhancement of the scattering cross-section, which is reported in some studies to be in the order of 10^1^–10^2^ is not considered in this equation as the electromagnetic enhancement expressed in Equation 5 dominates with enhancement factors of *G*(*hν*)^2^ = 10^10^.^3,43,105^ This is why the electromagnetic enhancement often plays the main role in the design of plasmonic nanostructures for SERS. The quadratic relationship between SERS and intensity enhancement emerges from the amplification of both the incident and scattered fields. The high SERS enhancement enables label-free detection of single molecules despite the very low Raman scattering cross sections.^43^ The Raman shifts are commonly measured in the VIS and NIR because of the availability of inexpensive optical components and well-established plasmonic materials like gold and silver in this spectral range.^47,106^ Moreover, the quadratic dependence on the field enhancement makes (sub)nanometer cavities and sharp tips ideal for maximizing SERS signals.

### Plasmon-Enhanced Infrared Absorption Spectroscopy

Infrared absorption spectroscopy enables the examination of vibrational states of a molecule by absorption measurements. Figure 1c shows how a molecule in its ground state can absorb photons that match their vibrational energy *hν* = *hν*_vib_ by transitioning into an excited vibrational state. These vibrational transitions manifest themselves as absorption lines in the spectral fingerprint region between λ = 6.67–20 μm. Infrared absorption spectroscopy also allows label-free detection of analytes and is complementary to Raman spectroscopy, as some vibrational transitions are exclusively infrared- or Raman-active. The power absorbed by an analyte
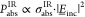
6is given by its infrared absorption cross-section, σ_abs_^IR^, and the intensity of the incident field, |*E̲*_inc_|^2^. Infrared absorption cross sections are typically around 10^–20^ cm^2^ and therefore significantly smaller than fluorescence absorption cross sections but well above Raman scattering cross sections.^52^ However, in contrast to SERS, plasmonic nanostructures only provide surface-enhanced infrared absorption (SEIRA) enhancements^104^
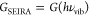
7that scale linearly with the intensity enhancement at the vibration energy, *hν*_vib_, and are typically between *G*_SEIRA_ = 10^3^–10^5^. Achieving single-molecule sensitivity with SEIRA has been hampered in part by the lack of adequate light sources and detectors in the MIR.^62,107^ Gold, graphene, or highly doped semiconductors offer LSPRs in this spectral range and are commonly used for SEIRA.^60^ Deep subwavelength gold nanoslits have demonstrated higher SEIRA enhancement to solid nanoantennas due to their higher intensity enhancement and increased detection volume allowing the detection of larger analyte volumes.^59^

### Plasmon-Enhanced Circular Dichroism

Enantiomers are molecules that cannot be superimposed with their mirror image through translation or rotation. The chirality, or handedness, of an enantiomer can decide whether a molecule has curative or adverse physiological effects and is therefore of great interest for the pharmaceutical industry.^108^ The asymmetry between right (R)- and left (L)-handed enantiomers can be optically differentiated through the circular dichroism
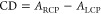
8shown in Figure 1d which is the differential absorption of left- and right-handed circular polarized (LCP and RCP, respectively) light. The CD enhancement factor^67^
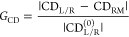
9is calculated from the CD_L/R_ and CD_L/R_^(0)^ of the L- or R-enantiomer in the presence and absence of a plasmonic nanostructure, respectively, and the CD_RM_ of a racemic mixture (1:1 mixture of both enantiomers) in the presence of a plasmonic nanostructure.

Three different mechanisms, nonmutually exclusive and not yet fully understood,^109,110^ have been explored for plasmon-enhanced circular dichroism sensing: (i) Creating chiral nanostructures from achiral nanoparticles (NPs). Fabrication methods include e-beam lithography (EBL), relying on the natural chirality of biomolecules such as DNA, peptides, proteins, micelles, etc., to generate scaffolds for the attachment of the NPs, or via direct growth during NP synthesis.^111−113^ Sensing is mostly based on the fact that the target analytes modify the chirality of the nanostructures, i.e., by contributing to their assembly or disassembly. (ii) Generating superchiral near-fields that strongly interact with chiral molecules to enhance the CD signals. Fabrication of superchiral structures typically involve challenging and expensive multisteps lithographic methods, in particular for 3D chiral geometries.^111^ Due to the complexity of fabrication and the fact that it is difficult to separate the contribution of the chiral nanostructures from that of the analyte to be investigated, biosensing applications based on superchiral nanostructures are still scarce. (iii) Via plasmon-coupled CD (PCCD), where the chiral molecule is placed in the *hotspot* of an achiral nanoantenna, inducing an intense chiral enhancement at the LSPR of the nanoantenna. Most reliable PCCD fabrication methods usually rely on DNA origami to construct antenna templates with various chiral analytes that can in principle be placed in the plasmonic *hotspot* for ultrasensitive chirality sensing.^111,112^ Interestingly, achiral gap nanoantennas have demonstrated superior performance over chiral ones in terms of CD enhancement, with enhancement factors of up to *G*_CD_ = 10^3^, underlining the importance of the intensity enhancement *G* provided in the plasmonic *hotspot.*(64,67) Finally, it is worth noting that although the absorption bands of many chiral analytes are in the UV,^86^ gold nanostructures are commonly used to enhance the CD in the VIS to NIR. This is partly due to the difficulty in detecting UV light and the limited availability of suitable plasmonic materials in this spectral range.^111^ Despite the high enhancement factors around the spectral LSPR band, the absolute CD can still be higher at the analyte’s absorption band in the UV.^67^ Thus, matching the LSPR and molecular absorption bands with plasmonic materials such as aluminum^86,111^ could enable the previously elusive goal of plasmon-enhanced chiral sensing with single-molecule sensitivity.

### Plasmon-Enhanced Refractive Index Sensing

The LSPR of plasmonic nanostructures is highly sensitive to refractive index changes, *∂n*, in their environment. This sensitivity can be used to detect analytes through their distinct refractive indices *n* + *∂n* by measuring the changes in the LSPR line shape, as illustrated in Figure 1e. LSPR-based refractive index sensors are typically assessed using the figure of merit^4^
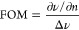
10which is the ratio between the sensitivity *S* = *∂ν*/*∂n*, indicating the LSPR frequency shift ∂ν = ν(*n* + *∂n*) – *ν*(*n*) per unit change in refractive index *∂n*, and the LSPR full-width half-maximum (fwhm) Δν. It has been noted that the FOM more accurately assesses sensing performance when it incorporates the LSPR modulation into its standard definition.^84,114,115^ Sensitivities of *S* ≈ 500 nm/RIU (refractive index unit) and FOMs of up to about 100 RIU^–1^ are reported for LSPR-based refractive index sensors.^76,82^ The high sensitivities *S* are achieved by confining fields of large magnitude in the analyte volume.^84,116^ On the one hand, this explains why subwavelength plasmonic *hotspots* are more suitable for refractive index sensing applications requiring high thin-film or single-molecule sensitivity than those requiring high bulk sensitivity.^7,117^ On the other hand, it underlines that the intensity enhancement described in Equation 1 plays a crucial role in improving the sensitivity *S* and consequently the FOM. Gold is a standard plasmonic material for refractive index sensing due to its relatively low Ohmic losses providing narrow line widths and its LSPR in the VIS to NIR where stable light sources and sensitive detectors exist. Plasmonic nanostructures that support subradiant modes are frequently used to minimize the LSPR line width by controlling radiative losses and thus maximize the FOM.^74,75,84,114^ The LSPR changes are usually detected by either (i) tracking the LSPR frequency shift using white light illumination and spectrometric detection,^78,81,118^ (ii) measuring changes in transmission or reflection using a narrow-band source and a photodetector,^7,10,85^ or (iii) detecting changes in the color of the nanoplasmonic sensor surface.^73,74,76^ Although refractive index sensing allows for label-free analyte detection, it often requires functionalizing the plasmonic nanostructures to ensure selective analyte detection.^7,73,81,85^

## CURRENT NANOPLASMONIC PLATFORMS FOR BIOSENSING *IN VITRO* AND *IN VIVO*

### Biosensing Based on Plasmon-Enhanced Fluorescence

**Figure 2:**
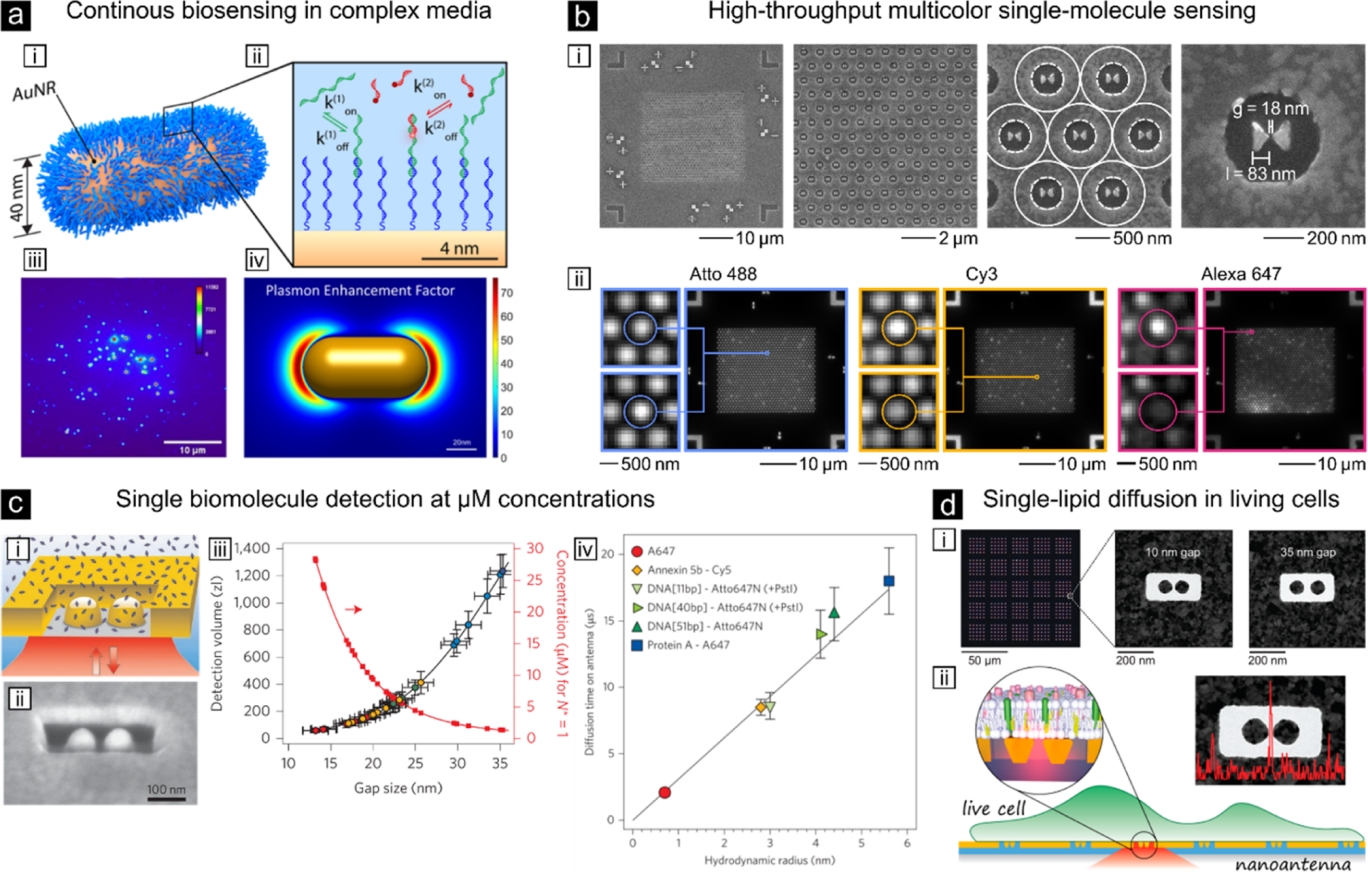
Examples of plasmon-enhanced fluorescence. (a,i–ii) Continuous single-molecule biosensing in complex matrices using gold nanorods (NRs) with low-affinity capture probes (blue) (i) and a sandwich assay with the analyte (green) and a low-affinity fluorescent label (red) (ii). (a,iii) wide-field fluorescence snapshot where each spot corresponds to a gold NR. (a,iv) simulations of the field enhancement for a gold NR of 40 nm in diameter with a longitudinal plasmon resonance at 640 nm. The weak affinity interactions facilitate continuous tracking of analytes with pM sensitivity over hours.^29^ Reproduced with permission from ref (29). Copyright 2024 American Chemical Society. (b,i) SEM images of hexagonal close-packed antenna-in-box arrays made of aluminum. (b,ii) High-throughput multicolor detection of three different fluorescence molecules (Atto488, Cy3 and Alexa647) detected quasi-simultaneously. The high-density packing enables capturing even very rare single-molecule events.^90^ Reproduced with permission from ref (90). Copyright 2024 American Chemical Society. (c,i–ii) Antenna-in-box platforms for detecting single biomolecules diffusing over the *hotspot* regions of the dimer antennas. (c,iii) Single-molecule detection as a function of the hotspot gap size, showing single molecule detection at elevated μM concentrations. (c,iv) Detection of different analytes by means of recording their diffusion times over the antenna hotspot.^121^ Reproduced with permission from ref (121). Copyright 2013 Springer Nature Limited. (d,i) Biocompatible plasmonic sensor based on arrays of antenna-in-boxes for studying the diffusion of individual lipids in the plasma membrane of living cells (ii).^15^ Reproduced with permission from ref (15). Copyright 2017 American Chemical Society.

As briefly mentioned, the intrinsic properties of fluorescence in terms of high absorption cross-section and quantum yield, selectivity, specificity, and biocompatibility make this contrast mechanism one of the most powerful methods for biosensing and live cell research. When combined with plasmonic enhancement the advantages multiply as both the absorption cross-section and the quantum yield can be increased, in particular, for weakly fluorescent dyes. Not surprisingly, a vast literature in the field has reported biosensing applications of a large number of analytes, including biomarkers, pathogens, and toxins, using plasmon-enhanced fluorescence assays (reviewed by Bauch et al.^119^). The incredible sensitivity of such approaches had also led to numerous applications down to the single molecule biosensing level, where both sandwiched or competitive assays have demonstrated detection levels down to the fM range in a few minutes.^120^ These types of assays combined with plasmonics have been recently reviewed by Dey et al., highlighting in particular the future challenges in the field in terms of miniaturization, integration, as well as those associated with real diagnosis applications requiring continuous biosensing.^29,120^ Along these lines, a major advance toward continuous detection of biomarkers has been recently reported by the Zijlstra Lab.^29^ In here, the authors cleverly relied on the weak affinity of unlabeled analytes binding to DNA-functionalized gold nanorods (NR) as well as the reversibly on–off binding of the fluorescently labeled probes (see Figure 2a). Impressively, using this strategy, the authors could perform repetitive measurements over hours using undiluted blood serum, without filtration or washing steps, and on a platform of hundreds of immobilized gold NRs probed in parallel, thus significantly increasing the detection throughput. A way to further increase the acquisition throughput while extending the detection of fluorescence to multiple colors has been reported by Herkert et al.^90^ The authors fabricated hexagonal close-packed aluminum-based plasmonic nanostructures using EBL fabrication (Figure 2b). This approach contains two major improvements over the immobilized gold-NR platforms described above. First, controlled hexagonal close-packing by EBL increased the number of plasmonic nanostructures and thus the single-molecule detection throughput by 1000-fold. Second, the use of aluminum extended the range of spectral detection to the full visible regime, so that multiple analytes labeled with different fluorescent dyes could be simultaneously detected.^90^ The combination of such engineered broadband platforms together with the weak-affinity sandwiched biosensing reported by the Zijlstra Lab could enormously extend the detection of thousands of different analytes in parallel.

While single biomolecule fluorescence detection at low sample concentrations can be readily performed using conventional optics and enhanced by the use of plasmonics, a major challenge that cannot be met by traditional optics is the detection of individual molecules at concentrations beyond the pM-nM range. Here, the strong light confinement and enhancement of plasmonic nanostructures are of particular relevance to the study of enzymatic reactions and/or biomolecule interactions that require high molecular concentrations.^17^ In a seminal paper, Punj et al. demonstrated that the small *hotspot* region between adjacent gold-dimer nanoantennas was sufficient to detect individual fluorescently labeled DNA samples at concentrations above 10 μM (Figure 2c).^121^ Such an approach was further improved to allow the detection of individual lipids and proteins in biological and fully intact living cell membranes at their endogenous levels (Figure 2d).^14,15^ More recently, the same authors combined the gold-dimer platforms with fluorescence correlation spectroscopy in a wide field configuration to detect the diffusion of individual proteins in the membrane of living cells in a high throughput fashion.^122^ More challenging but exciting applications relying on plasmon-enhanced fluorescence are also emerging for *in vivo* imaging with a recent demonstration of gold-NRs to enhance short-infrared upconversion emission intensity of fluorescent dyes (around 1000 nm), and being capable for the first time of *in vivo* detection of ovarian cancer in mice with high sensitivity.^123^

### Biosensing Based on Plasmon-Enhanced Raman Scattering

In contrast to fluorescence, Raman signals do not suffer from photobleaching and are specific to the biomolecule to be detected avoiding the need for labeling. Importantly, the intrinsic sharpness of vibrational bands allows multiplexing the spectroscopic information to readily allow discrimination between different reporter and analyte molecules. Unfortunately, the major limitation of standard Raman spectroscopy is the extremely weak signals obtained, requiring in turn long integration times.

**Figure 3:**
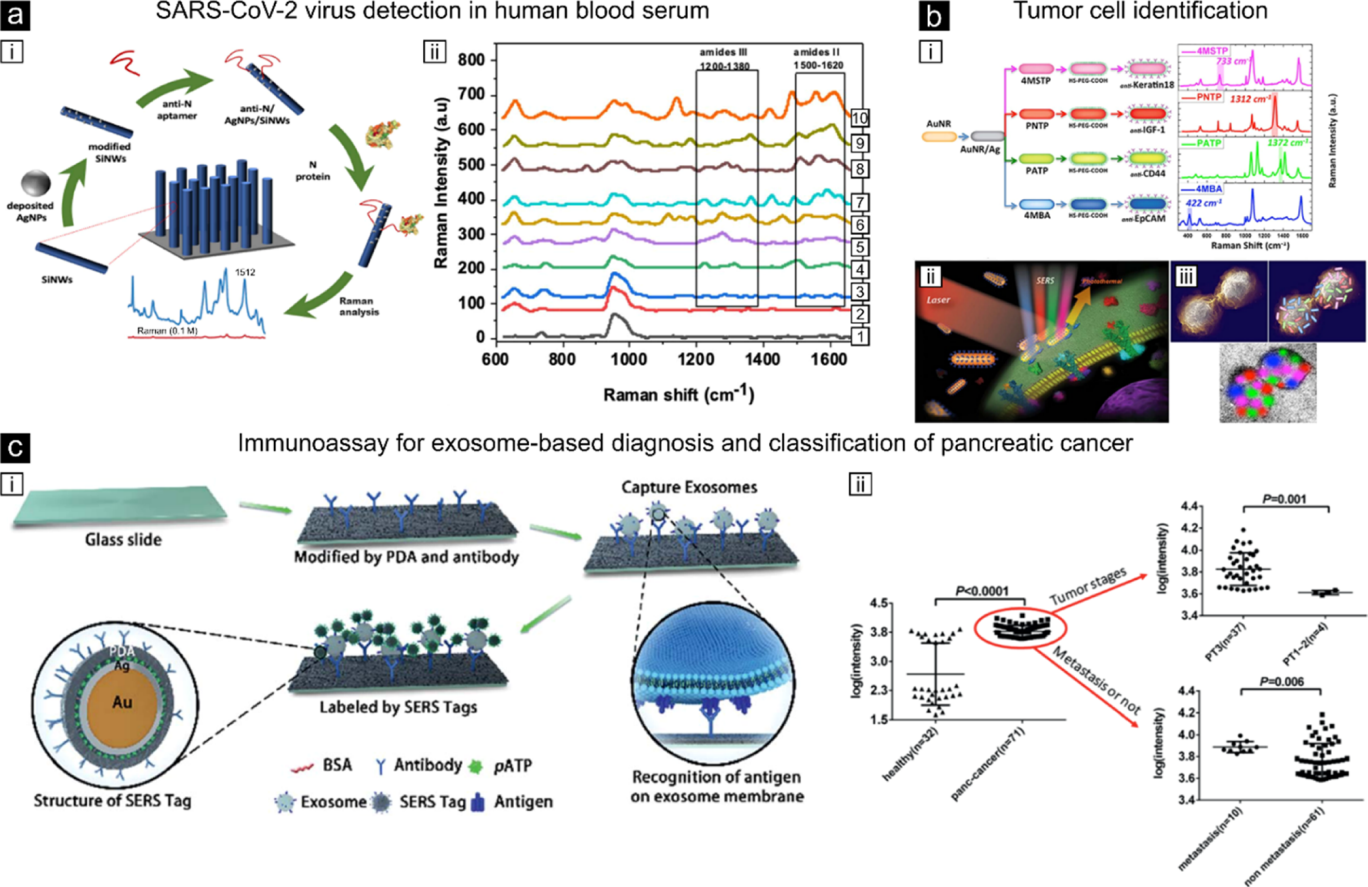
Examples of plasmon-enhanced Raman scattering. (a,i) Illustration of the coronavirus nucleocapsid protein detection steps using silver-NPs covered on silicon nanowires. (a,ii) Acquired SERS spectra at increasing protein concentrations, from 1 ng/mL to 75 ng/mL (spectra labeled from 3–10).^126^ Reproduced from ref (126). Available under a CC-BY 3.0. Copyright 2024 The Royal Society of Chemistry. (b,i) Scheme of the multiplexed detection of four different cancer biomarkers using specific antibodies after functionalization of gold–silver NRs using different SERS nanotags (left) and assigned to four nonoverlapping spectral peaks (right). (b,ii) Sketch of the four biomarkers targeting the breast cancer cell surface. (b,iii) Illustration of the 2D multicolor SERS data with recognition of four different cell surface receptors on breast cancer cells.^128^ Reproduced with permission from ref (128). Copyright 2014 Springer Nature Limited. (c,i) Scheme of the SERS immunoassay using a polydopamine (PDA)- and antibody-modified glass slide, capture exosomes, and polydopamine-encapsulated antibody-reporter-Ag(shell)–Au(core) multilayer (PEARL) nanotags. (c,ii) Logarithmic SERS intensities from serum samples of pancreatic cancer patients and healthy controls.^129^ Reproduced with permission from ref (129). Copyright 2018 The Royal Society of Chemistry.

As described above, SERS via plasmon-enhanced coupling can enhance Raman signals by orders of magnitude. Because of this, biomedical applications of SERS with increasing medical significance have grown exponentially in the past decades. Such applications span the detection of different types of analytes or biomarkers, including those for earlier cancer detection, viral and bacterial detection, the monitoring cardiovascular and neurodegenerative diseases as well as for the monitoring of drug delivery, among many others. Most of these applications have been extensively reviewed in several outstanding contributions.^49,124,125^ We therefore restrict in here to few prominent examples from the literature that aim to push SERS to applications involving a higher level of biological complexity and/or versatility in terms of biosensing and biodetection (see Figure 3). Biological complexity refers to the detection of biomolecules or analytes in relevant biofluids without filtration, i.e., urine or blood serum, and SERS signals in the context of living cells and tissues. A notable example of the first is the recent detection of the SARS-CoV-2 virus in real samples of human blood serum with an impressive detection limit of 1 ng/mL using an aptamer-based SERS sensor based on silver NPs decorating silicon nanowires (Figure 3a).^126^

In terms of living cells and tissues, SERS has been used to detect different biomolecules inside cells, i.e., intracellular SERS, with examples that include the detection and tracking of specific drugs, cancer biomarkers, metabolites, targeting specific intracellular compartments, and monitoring cellular functions.^37,38,127^ Nevertheless, intracellular SERS applications continue to be highly challenging and prone to artifacts, basically due to the larger size of the NPs as compared to fluorescent labels (50–60 nm for SERS, 2–4 nm for fluorescence). This major drawback difficult the efficient and controlled uptake of NPs through the cell membrane, requiring specific methods for the appropriate internalization of the NPs without interfering with the integrity of the membrane and/or intracellular processes.^127^ Moreover, NP delivery to specific intracellular compartments can be problematic as NPs tend to accumulate in vesicles and to follow the endolysosomal pathway for their degradation. Therefore, strategies that include NP surface functionalization toward the specific intracellular compartments should be considered. Finally, NPs tend to aggregate inside cells affecting not only the NP properties in terms of SERS signals but also importantly, compromising cellular processes and/or exacerbating cell toxicity, as metal NPs are known to produce oxidative stress leading to cell death.^49,127^ In contrast, one of the most reliable and powerful applications in our opinion is the use of SERS nanotags for selective protein recognition on the cell surface, i.e., immuno-SERS labeling, being analogous to immuno-fluorescence labeling. In this approach, a Raman-reporter molecule is attached to the NP for signal enhancement and a targeting moiety (usually an antibody for protein recognition) is used to direct the nanotag to the specific target protein allowing its indirect detection through the enhanced SERS signal of the Raman-reporter. Usually, a protective coating is used for both stabilization and for grafting of the targeting moiety. Because antibody recognition is highly specific to the protein of interest, SERS nanotags can be used for highly multiplexed detection of different proteins in cells. Moreover, as the targeted proteins are expressed on the cell surface, NP labeling is much more amenable and less prone to artifactual labeling. One of the earliest applications of such concept, still considered as the gold-standard demonstration in the field, constituted the detection of four different breast cancer cell markers in unprocessed human blood (see Figure 3b).^128^ For this, the authors relied on hybrid silver–gold NRs conjugated to different Raman nanotags and to four different antibodies specific for cancer cell identification, reaching discrimination of different types of breast cancer cells with impressive specificity (down to a single cancer cell within millions of blood cells). More recent cutting-edge developments are pushing the field toward the implementation of SERS immunoassays to discriminate different types of cancers from clinical serum of human patients (Figure 3c)^129^ or on 3D tumoral models (thoroughly reviewed by Troncoso-Afonso et al.).^130^

### Biosensing Based on Plasmon-Enhanced Infrared Absorption Spectroscopy

IR absorption spectroscopy is a powerful technique that directly measures the vibrational modes of molecular bonds in the mid-infrared region (typically between 3 and 20 μm) and thus, in principle, provides chemical specificity in a nondestructive and label-free manner. As discussed above, by relying on plasmonic nanostructures, the low IR absorption cross-section of biomolecules can be significantly enhanced, which has led to the emergence of surface-enhanced infrared absorption (SEIRA) techniques. Yet, the overlap of water absorbance bands with that of proteins at this spectral range has prevented the extended use of SEIRA in life science applications. This bottleneck was addressed in 2013 by the Altug group by developing a plasmonic internal reflection (PIR)-SEIRA concept by which only the axial extent of the evanescent field is used to excite the sample, reducing dramatically signals from the bulk water absorption bands.^131^ This major achievement has increased SEIRA applications toward the detection of different biomarkers *in situ* in combination with microfluidic approaches,^62,131,132^ although real applications are still rare in the literature.

**Figure 4:**
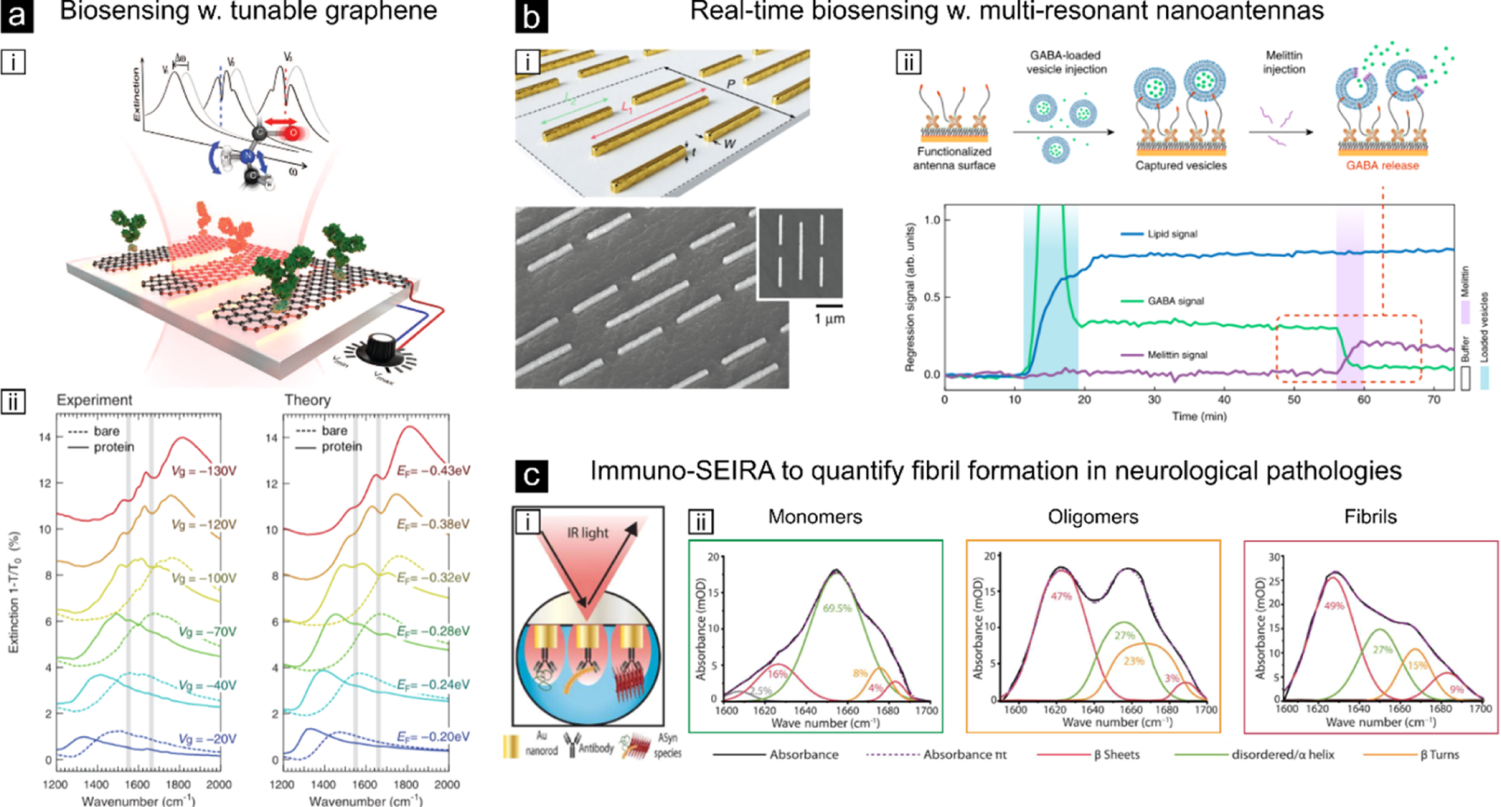
Examples of plasmon-enhanced infrared absorption spectroscopy. (a,i) Schematic of the dynamically tunable graphene MIR biosensor. (a,ii) Experimental and theoretical spectral tunability of the graphene nanoribbon array extinction spectra through electrostatic gating before (dashed lines) and after (solid lines) protein bilayer formation. The gray vertical areas indicate the amide I and II vibrational bands of the protein.^53^ Reproduced with permission from ref (53). Copyright 2015 American Association for the Advancement of Science. (b,i) Rendering and scanning electron microscopy image of a multiresonant plasmonic sensor based on superimposed NRs arrays with different dimensions. (b,ii) Illustration of the real-time vesicle cargo release experiment and time-resolved linear regression signals from spectral data of lipids (vesicle), GABA (cargo), and melittin (pore protein).^133^ Reproduced from ref (133). Available under a CC-BY 4.0. Copyright 2018 Springer Nature Limited. (c,i) Immuno-SEIRA detection principle where specific antigens bound to gold-NRs recognize α-synuclein proteins involved in Parkinson and Alzheimer diseases. (c,ii) Conformational profiling of different α-synuclein protein species allowing quantification of the percentage of monomers, oligomers, and fibrils.^134^ Reproduced with permission from ref (134). Copyright 2023 The American Association for the Advancement of Science.

A prominent demonstration by the Altug and Pruneri groups relied on the fabrication of a graphene-based tunable MIR sensor to detect the vibrational fingerprints (as well as refractive index changes) of proteins immobilized on the sensor (Figure 4a).^53^ The authors convincingly showed that graphene increased the detection sensitivity of the amide I and II bands of the proteins as compared to gold-based SEIRA sensors and most importantly, extended the spectral band for detection due to the dynamic tunability of graphene. A variation of this work by using multiresonant plasmonic structures instead of exploiting the dynamic tunability of graphene demonstrated simultaneous 1000-fold enhancement of the near-field over different spectral bands (Figure 4b).^133^ The multiresonance character of such sensor was cleverly achieved by the superposition of two different types of dipole arrays having different dimensions. In this way, the spectral response contained two marked resonances spectrally separated from each other and tuned to selectively enhance the amide and methylene absorption bands. Such an approach enabled for the first time to resolve interactions between mimetic lipid membranes with different polypeptides in real time. Remarkably, the authors succeeded in monitoring in real time GABA release (a neurotransmitter protein) upon the action of melittin (a pore-formation protein) in mimetic vesicles (see Figure 4b). More recently, the same group combined an optofluidic SEIRA sensor based on gold-NR arrays with antibody functionalization to detect and robustly quantify different structural species of the α-synuclein protein associated with several neurodegenerative pathologies such as Parkinson and Alzheimer diseases (see Figure 4c).^134^ Impressively, by incorporating artificial intelligence in the analysis of the detected SEIRA signals, the authors discriminated different ratios of α-synuclein monomers, oligomers and fibrils (the latter two being associated with these diseases) in human cerebrospinal fluids. Moreover, as a proof-of-concept, they functionalized the arrays using two different antibodies against α-synuclein and the tau-proteins (also associated with these disorders), constituting a first step toward multiplexed biomarker detection by means of SEIRA. While these examples are truly exceptional demonstrating the potential of the technique, there are several challenges that need to be overcome before SEIRA finds a widespread use in clinical applications. Some of these challenges include the lack of reproducibility of the SEIRA signals due to instabilities of the SEIRA metal substrates which are sensitive to oxidation or corrosion under physiological conditions used for bioanalyte detection. The resultant variations of the LSPR of the SEIRA substrate and of the interactions with the bioanalyte affect both sensing capability and reproducibility. Additional factors such as overlapping infrared peaks, interference signals and the reduced availability of highly sensitive detectors in the MIR region can significantly impair the performance of SEIRA for bioanalyte detection.

### Biosensing Based on Plasmon-Enhanced Circular Dichroism

Chirality is a ubiquitous property of most biomolecules and abnormal enantiomeric ratios of chiral molecules have been found in multiple pathologies, including cancers, and kidney and brain diseases.^135^ Because of this, chiral molecules have the potential to become excellent biomarkers for disease diagnosis and prognosis, but also to monitor drug effects on patients in a personalized manner. Unfortunately, reliable detection of chiral molecules for clinical applications is quite challenging due to their large variation in terms of chemical properties, their small size and low presence in biofluids and tissues (ranging from pM to nM). This is exacerbated by the fact that standard techniques to detect CD signals, such as chromatography, capillary electrophoresis, standard chiral spectroscopy methods, etc., suffer from limited sensitivity, in the μM to mM range, well below the sensitivity window required for selective molecular chiral detection.^135^

As mentioned above, biosensing of chiral molecules based on plasmon-enhanced circular dichroism offers enormous potential to enhance CD signals by orders of magnitude as compared to standard CD detection approaches. We highlight in here only a few examples of what we believe are the most striking and relevant applications with clinical potential while the reader is referred to excellent reviewers in the field summarizing efforts toward plasmon-enhanced circular dichroism sensing of a variety of other biomolecules.^110−112,136^

**Figure 5:**
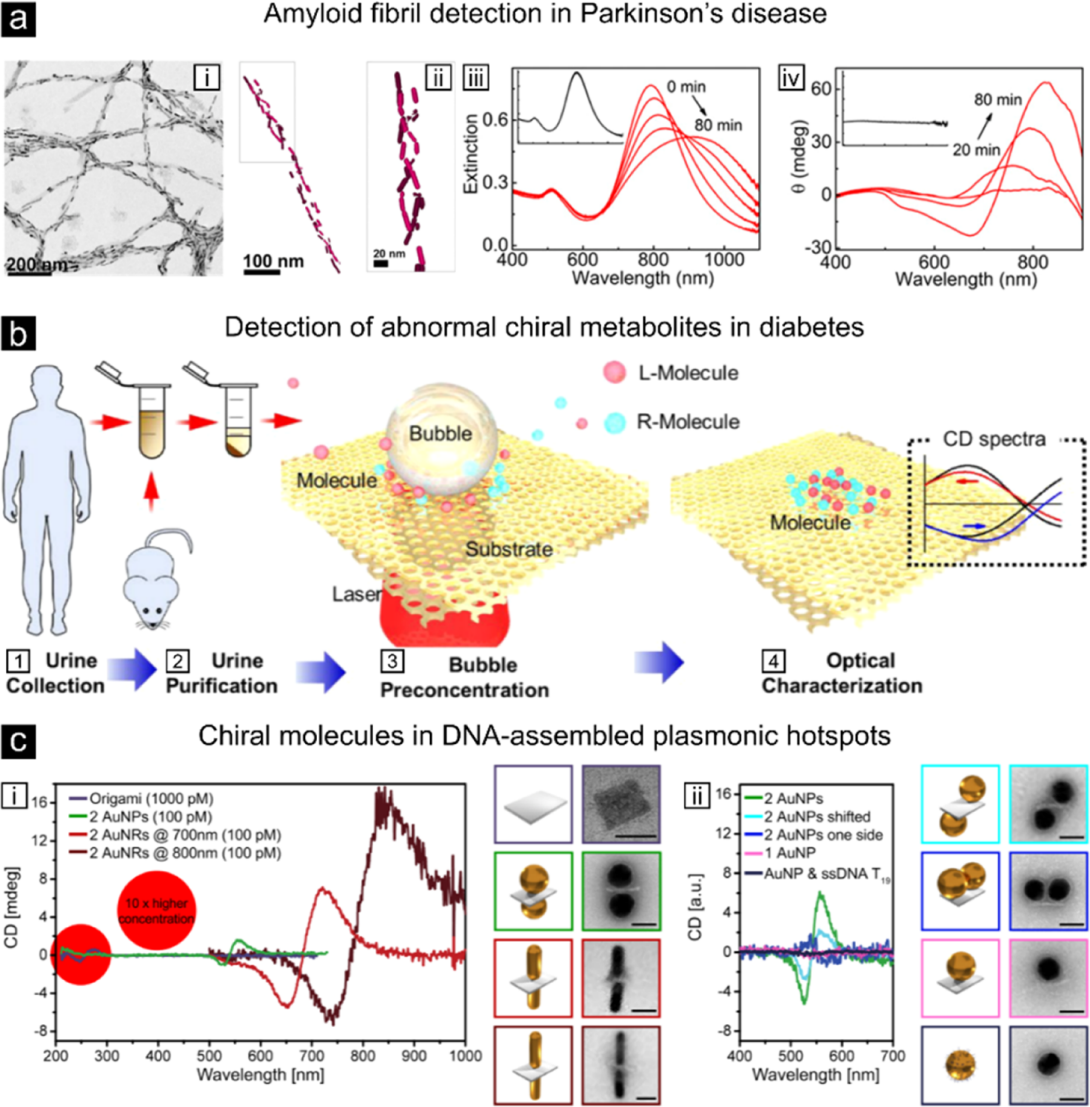
Examples of plasmon-enhanced circular dichroism. (a,i) Transmission electron microscopy (TEM) images of 0.5 nM of gold NRs in the presence of α-synuclein fibrils. (a,ii) 3D cryo-TEM reconstruction showing the 3D chiral NR arrangement. (a,iii) Extinction and (a,iv) CD spectra up to 80 min after the additions of the α-synuclein fibrils. Insets show the absent changes in CD spectra when adding α-synuclein monomers.^137^ Reproduced with permission from ref (137). (b) Illustration of the collection, purification, enhanced concentration of the analyte, and enhanced CD spectroscopy steps using plasmonic Moiré chiral metamaterials for chiral metabolite detection (shown here for glucose).^139^ Reproduced with permission from ref (139). Copyright 2021 American Chemical Society. (c,i) CD for an isolated chiral DNA origami structure at 1000 pM and chiral DNA origami structure at 100 pM with gold nanoparticles (AuNPs), nanorods resonant at 700 nm (AuNRs @ 700 nm), and NRs resonant at 800 nm (AuNRs @ 800 nm) attached. AuNRs @ 800 nm exhibit up to 300-fold enhancement. (c,ii) CD for different AuNP arrangements.^141^ Reproduced with permission from ref (141). Copyright 2018 American Chemical Society.

An impressive application with clinical potential has been reported by the Liz-Marzán Lab,^137^ where the authors succeeded on the detection of α-synuclein amyloid fibrils associated with Parkinson disease by relying on the structural chirality provided by the amyloid fibrils (Figure 5a). Whereas gold-NRs showed no preferred interaction with α-synuclein monomers, they aligned helically on amyloid fibrils and exhibited strong chiroptical response that the authors then used for nM sensitive detection of amyloid fibrils, both *in vitro* and extracted from brain homogenates of patients affected by Parkinson disease.^137^ In an additional work, the same group relied on DNA chirality to intracellularly self-assemble gold-NRs, whose assembly (and thus CD signal) depended on the presence of microRNA (miRNAs) inside living cells. Remarkably, the authors demonstrated sensitivity down to detection of a single base-pair mismatch of miRNA, i.e., fM/10 μgRNA, underscoring the potential of such sensors for real-time miRNA detection in living cells in applications involving monitoring disease progression, with outstanding selectivity and sensitivity.^138^

Superchiral near-fields have been also used for highly sensitive detection of clinically relevant chiral molecules. An excellent example is the accumulation-assisted plasmonic chiral sensing of glucose and lactate at concentrations down to 100 pM.^139^ This work takes advantage of superchiral near fields generated by plasmonic moiré chiral metamaterials together with increased molecular occupation using microbubbles. The authors further used this approach to uncover diabetes-induced abnormal chirality in mice and human urine with a high diagnostic accuracy of 84% for human clinical samples (Figure 5b).^139^ More recently, superchiral near-fields with selective enhancement of single-handed superchiral fields have been engineered and used in combination with vibrational CD (VCD) in the MIR to detect thalidomide, a drug with severe adverse effects depending on its structural chirality, but increasingly used for cancer treatment and other pathologies.^140^ The authors showed 13-orders of magnitude enhancement on the VCD dissymmetry factors as compared to standard VCD, in concentrations as small as 50 μM (standard VCD requires typically mM concentrations).^140^

Finally, the potential of plasmon-coupled CD is large in particular toward single analyte detection. Most efforts so far has been devoted to understand and accurately control the distance separation between dimer nanoantennas, i.e., the size of the *hotspo*t region for maximum plasmon coupling of chiral molecules located at the *hotspot*. An elegant demonstration of such effect was reported by Kneer et al. using DNA origami of different lengths (Figure 5c).^141^ The authors showed up to 300-fold enhancement of the CD signal at the local plasmon resonance of gold-NRs as compared to the characteristic UV CD of DNA at analyte concentrations as low as 100 pM. Although highly promising for the ultimate goal of single-molecule chiral detection, true applications in the relevant biosensing and clinical settings are yet to come.

### Biosensing Based on Plasmon-Enhanced Refractive Index Changes

**Figure 6:**
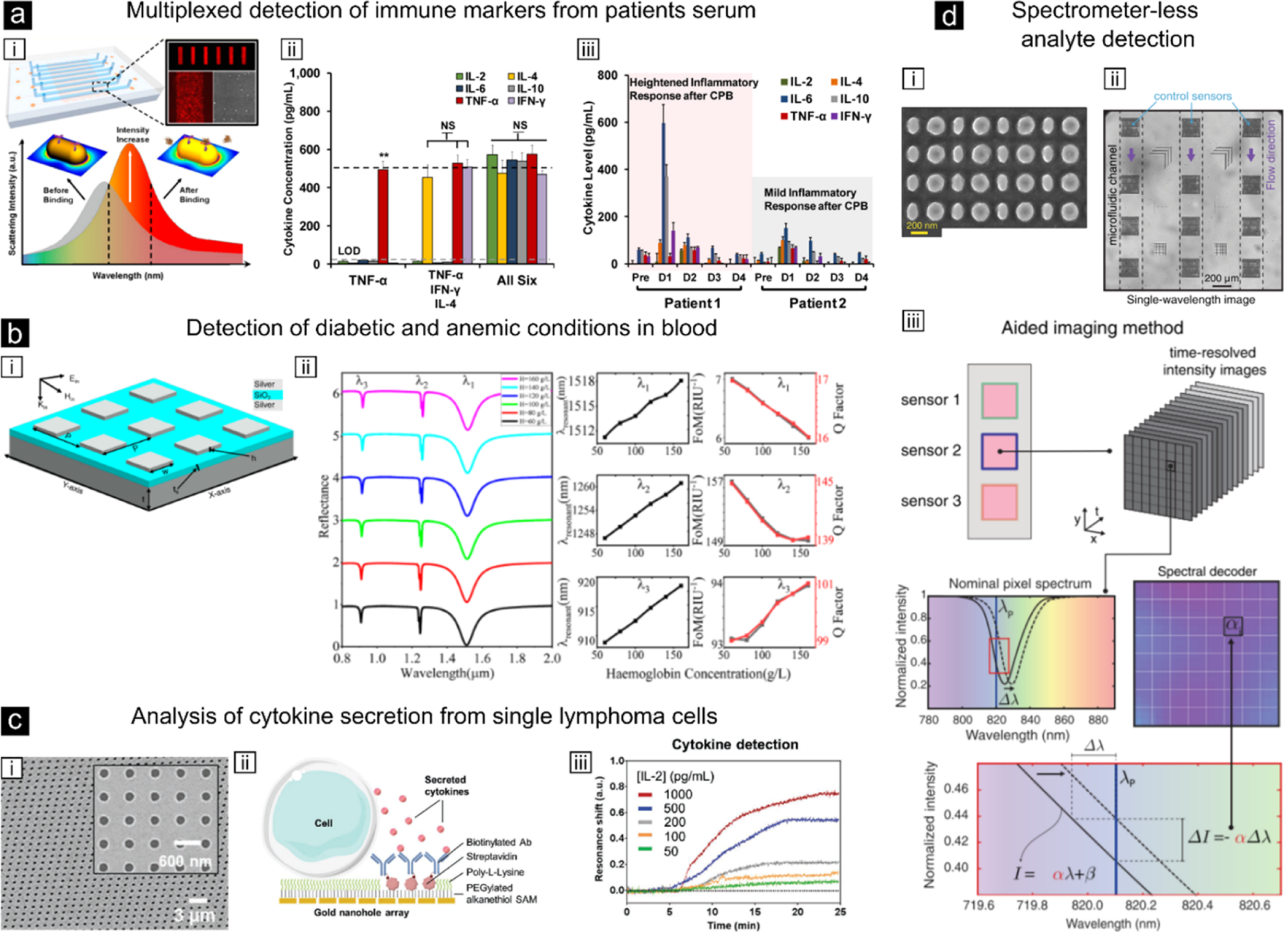
Examples of plasmon-enhanced refractive index sensing. (a,i) Scheme of the plasmonic microarray integrated into eight parallel microfluidic detection channels. Binding of the analyte induces an LSPR redshift and increase of the scattering intensity. (a,ii) Quantified cytokine concentrations for different sample mixtures of recombinant cytokines in serum. (a,iii) Cytokine concentration variations for serum extracted from two cardiopulmonary bypass (CPB) surgery patients measured for 5 days with the plasmonic microarray sensor.^146^ Reproduced with permission from ref (146). Copyright 2015 American Chemical Society. (b,i) Sketch of the metal-dielectric-metal metasurface used for refractive index sensing. (b,ii) Simulations of the spectra containing three distinct resonances of the metasurface and their respective wavelength shifts, FOMs, and Q-factors for the detection of different hemoglobin concentrations in blood. Analogous simulations have been done for detection of glucose in blood.^147^ Reproduced with permission from ref (147). Copyright 2024 American Chemical Society. (c,i) Scanning electron microscopy image of the plasmonic gold nanohole array. (c,ii) Functionalization of the gold surface for cell attachment and in situ detection of secreted cytokines by individual lymphoma cells. (c,iii) Real-time plasmonic resonance shift for different cytokine (IL-2) concentrations.^149^ Reproduced with permission from ref (149). Copyright 2018 Wiley. (d,i) Scanning electron microscopy image of a dielectric metasurface. (d,ii) Single-wavelength optical image of the optofluidic chip containing 3 × 4 of these metasurfaces. (c,iii) A spectral decoder allows spectrometer-free reconstruction of the wavelength shifts using the normalized intensity changes and a spectral decoder that is unique for each metasurface (sensor). This reconstruction can increase the signal-to-noise ratio and consequently the limit of detection.^150^ Reproduced from ref (150). Available under a CC-BY 4.0. Copyright 2021 Springer Nature Limited.

While a large number of studies have reported the detection of biomarkers based on refractive index changes in buffer solutions with outstanding limits of analyte detection (down to 100 fM for nucleic acids and 1 pg/mL for proteins),^142−145^ only a few studies so far have demonstrated detection of relevant analytes in complex biofluids (blood, urine, saliva, etc.) and/or showed applications in living cells. One of the first demonstrations of refractive index sensing in human serum samples for clinically relevant biomarker detection was reported by Chen et al. using gold-NR arrays arranged in stripes and combined with microfluidic channels (Figure 6a).^146^ The gold-NR stripes were functionalized with specific antibodies recognizing six different types of cytokines, proteins that report on the onset of inflammatory responses. Using this approach the authors succeeded in real-time multiplexed detection of six different cytokines with a limit of detection below 20 pg/mL from 1 μL of serum samples (Figure 6a). Importantly, the authors also demonstrated the ability to monitor over time inflammatory responses of patients following cardiopulmonary bypass surgery, underscoring the potential of such chip devices in clinically relevant settings.

Over the years, researchers have explored other materials aside from metals (in particularly of gold) to overcome their main limitation in terms of lower quality factors due to ohmic losses, and the restricted spectral range of increased sensitivity detection. A recent study of an hybrid plasmonic platform based on metal-dielectric-metal metasurfaces has been reported to combine the advantages of silver in terms of enhancement and thus sensitivity, and that of dielectrics in terms of increased quality factors (Figure 6b).^147^ Using FDTD simulations, the authors showed the emergence of three modes with different sensitivities at wavelengths in the NIR which could be used for quantitative detection of glucose and hemoglobin in blood, thus predicting concentration ranges for the detection of diabetic (i.e., glucose levels) and anemic (i.e., hemoglobin levels) conditions. This work illustrates the potential of hybrid sensors toward quantitative diagnosis of pathological conditions, awaiting now for their experimental demonstration, miniaturization and integration toward widespread use in the clinics. Approaches that combine gold-NRs together with graphene are also being pursued in an effort to improve the detection performance for ultrasensitive detection of analytes due to the coupling of evanescent fields on graphene and the LSPR of gold-NRs.^148^ Convincing applications of these type of sensors are still to come.

In the context of single cell analysis, gold nanohole arrays in combination with microfluidics have been devised to detect real-time cytokine secretion from individual lymphoma cells (Figure 6c).^149^ Microfluidics enabled to concentrate the minute amount of released cytokines by the cells while placing the molecules in closed proximity to the nanoholes for their sensitive detection. Using this highly complex optofluidic nanohole-based sensor, the authors reported the continuous measurement of the interleukin-2 cytokine secreted by individual cells over the course of 3 h with an excellent limit of detection of 39 pg/mL. More recently, the same group exploited an all-dielectric metasurface chip integrated in a optofluidic system for real time detection of extracellular vesicles from breast cancer cells (Figure 6d).^150^ Importantly, this work relied on the use of a single wavelength illumination and an advanced algorithm working as “spectral decoder” of CMOS camera data collected over a large field of view in real time, avoiding standard spectroscopy detection. In this way, the authors succeeded in considerably reducing the instrumentation needed, thus a first step toward the miniaturization of biosensors for point-of-care diagnoses purposes.

## CHALLENGES AND OPPORTUNITIES

The few selected examples described in the previous section and accompanying Figures 2–6 highlight the enormous advances in the field of plasmonic-enhanced biosensing achieved in recent years. Most of these examples showed the successful detection of specific biomarkers in complex biofluids (also known as liquid biopsies) by relying on the enhancement of specific molecular properties (electronic or vibrational transitions, chirality, and refractive index). However, most applications so far have relied on the enhancement of one specific molecular property, for instance, fluorescence or Raman signals, by tuning the fabrication and/or material properties of the plasmonic nanostructures to the spectral region of the given molecular property to be enhanced. Yet, for many clinical applications or relevant live cell studies, different biomolecules exhibiting distinct molecular fingerprints would have to be simultaneously detected for the unequivocal diagnosis of a given pathological condition (for instance in the cases of cancer, neurological and immunological disorders, among others). Instead of performing serial detections of different biomarkers using different plasmonic sensors, one could envisage the design of smart plasmonic nanostructures with multiple resonances at different spectral windows, and combined with narrow and broad resonances. This would enable the combination of different contrast mechanisms, ultimately leading to high-throughput multiplexed detection with a single sensor. Since contrast mechanisms like fluorescence, Raman scattering, refractive index, and circular dichroism are all probed within similar spectral ranges (VIS-NIR) and primarily benefit from plasmonic field enhancement, they can potentially be integrated into a single plasmonic sensing platform using the same plasmonic nanostructures, light sources, and detectors. Some emerging efforts are already pointing toward this direction with the simultaneous detection of enhanced fluorescence and SERS signals with the same sensor,^151,152^ the fabrication of chiral metamaterials that selectively amplify Raman signals that are sensitive to either left- and right-handed circularly polarized light,^153^ multimodal detection of SEIRA and SERS signals,^154^ and other multiple-signal-sensing mechanisms that can be combined with SERS, thoroughly reviewed by Langer et al.^49^ In particular, the narrow spectral lines of Raman signals make SERS extremely appealing for multiplexed detection of many different analytes^155^ that can be even combined with other contrast mechanisms to extend the detection range. Yet, one would need to carefully control the functionalization of the nanostructures as cross-reactivity and aggregation of antibodies, commonly required for immuno-SERS and immune-fluorescence based assays, can increase considerably at high surface concentrations.

Optofluidic and optoelectronic integration, miniaturization, cost-effectiveness, surface functionalization, and sample handling are some of the practical and current challenges to bring nanophotonics into the true realm of biosensing. Many of these challenges are being tackled by multiple groups around the world. In particular, the Altug Lab has shown outstanding proof-of-concept studies addressing optofluidic integration, cost-effective fabrication of different plasmonic nanostructures, and efforts toward miniaturization by replacing expensive and bulky instrumentation with artificial intelligence algorithms.^4,150,156^ There are recent reviewers in the field that address these challenges and the reader is referred to them for further insights into emerging approaches that can solve (at least partially) some of the current bottlenecks in the field.^4,49,120^

Compared to the detection of biomarkers in complex biofluids, studies in the context of living cells, in particular, measuring biomolecular interactions, are lacking behind for obvious reasons related to the complexity and molecular crowded environment inside cells. The need for advancing the technologies for live cell research is urgent as many relevant biomolecules are not present in body fluids. The same holds true in tissue research, in particular for the detection of cancer biomarkers in tumor tissues.^130^ Biosensing at the level of living cell membranes using plasmon-enhanced fluorescence^15,122,128,157,158^ and SERS has been accomplished,^15,122,128,157,158^ but obtaining relevant information regarding real-time interactions between different proteins or lipid–protein interactions are significantly more challenging since they require the detection of multiple signals simultaneously, in a dynamic fashion, over different regions of the cell and over multiple cells to overcome cell heterogeneity. To meet these stringent requirements, highly packed plasmonic arrays, broadband response, high-throughput readout, multiplexing capabilities, readout speed, biocompatibility, and optofluidic integration are all needed.

Even more complex is the detection of specific biomarkers inside cells for intracellular sensing and intracellular diagnosis. Although some examples have been shown in the literature using SERS-nanotags with the potential for multiplexed biomarker detection (reviewed by Langer et al.^49^), the success and reproducibility of these studies are in general poor and prone to artifacts. We already mentioned some of the challenges associated with intracellular SERS, mainly due to the size of the required NPs for signal enhancement. In parallel, the revolution in the fluorescence microscopy field brought about by the invention of super-resolution approaches,^159^ has led to an accelerated development for new fluorescent probes and labeling strategies for highly selective intracellular labeling and molecular monitoring. Halo- and SNAP-tag strategies allow nowadays the selective binding of bright fluorescent molecules to specific intracellular compartments^160−162^ while fluorophores are able to penetrate into the internal cell compartments without aggregation issues or vesicle accumulation, in contrast to most SERS nanotags. Environmentally sensitive fluorescence probes (measuring pH or calcium levels)^163,164^ have been also developed with excellent performance in terms of fluorescence photon emission without the requirement for further enhancement. Thus, while for some intracellular applications plasmonic nanostructures might be highly beneficial to achieve enhanced detection sensitivity, one would need to carefully evaluate other conventional approaches that could enable intracellular biomolecule detection (albeit with lower sensitivity), but in turn, could reduce operational complexity of plasmonic nanostructures and/or potential artifacts to the biological processes to be studied.

## Conclusions

In this review we have concisely overviewed the main physical concepts that lead to plasmon-enhancement of distinct molecular properties toward their enhanced sensitivity for biosensing applications in clinically and biologically relevant scenarios. Using specific examples from the literature, we have described different plasmonic biosensor platforms based on other materials aside from traditional metals such as gold, silver, or aluminum, with emergent developments using hybrid (combination of different metals, or metal and dielectrics), or metal–graphene metamaterial designs, among others. We have briefly explained prominent applications that relied on the enhancement of fluorescence, Raman scattering, infrared absorption, circular dichroism, and refractive index changes, for the selective and sensitive detection of different types of analytes. Given the rapid evolution of the field, we have focused on promising proof-of-concept studies that demonstrate the reliable and sensitive detection of clinically relevant analytes in complex biofluids for “liquid biopsy” diagnosis and for the monitoring of disease progression. Moreover, we highlight cases of plasmon-enhanced biomolecular sensing in the context of living cells or for identification of cancer cells in tissue, underscoring the current challenges associated with the increased complexity of such studies. While there is still room for new concepts that increase even further the sensitivity of such devices down to ultimate level of single bioanalyte detection, we believe that the challenges ahead are more directed to their robust translation to the clinics. Recent efforts are already pointing toward this direction and we have underlined some of them in terms of multiplexed, multiple-signal sensing, higher throughput, miniaturization, or integration. Yet, only by combining expertise and true collaborations between physicists, engineers, surface scientists, biochemists, biologists, and clinicians will the field of plasmon-enhanced biosensing be adopted reliably and routinely by the pharmaceutical and medical sectors.
